# Effects of Physical Activity and Micronutrients on Cognitive Performance in Children Aged 6 to 11 Years: A Systematic Review and Meta-Analysis of Randomized Controlled Trials

**DOI:** 10.3390/medicina58010057

**Published:** 2021-12-30

**Authors:** Atiah Munirah Meli, Asma’ Ali, Abbe Maleyki Mhd Jalil, Hayati Mohd Yusof, Michelle M. C. Tan

**Affiliations:** 1 Department of Food Science, Faculty of Fisheries and Food Science, Universiti Malaysia Terengganu, Kuala Terengganu 21030, Malaysia; atiahmeli97@gmail.com (A.M.M.); hayatimy@umt.edu.my (H.M.Y.); 2 School of Nutrition and Dietetics, Faculty of Health Sciences, Universiti Sultan Zainal Abidin, Kuala Terengganu 21300, Malaysia; abbemaleyki@unisza.edu.my; 3 Global Public Health, School of Medicine and Health Sciences, Monash University, Subang Jaya 47500, Malaysia; michelle.tan1@sydney.edu.au; 4 South East Asia Community Observatory (SEACO), Monash University, Subang Jaya 47500, Malaysia; 5 Victorian Heart Institute, Monash University, Clayton 3168, Australia; 6 Charles Perkins Centre, Central Clinical School, Faculty of Medicine and Health, The University of Sydney, Sydney 2006, Australia

**Keywords:** physical activity, micronutrients, cognitive performance, children

## Abstract

*Background and Objectives*: Cognitive performance is essential for children, given this is a critical stage of brain growth and development. This systematic review and meta-analysis aimed to ascertain if physical activity or micronutrients impact cognitive performance in children. *Materials and Methods*: Electronic databases (PubMed and Scopus^®^) were searched for relevant articles published between 2012 and 2021. We emphasized randomized controlled trials (RCTs) that examined the effect of physical activity and micronutrients on cognitive performance. Data from eligible studies were gathered and evaluated using random-, fixed- or pooled-effects models with 95% confidence intervals (95% CI). *Results*: Physical activity appeared to improve both Mathematics (*d* = 1.12, 95% CI: 0.56, 1.67) and attention (*d* = 0.65, 95% CI: 0.15, 1.14) performances. The micronutrient vitamin B12 had a positive effect on Mathematics (*d* = 2.39, 95% CI: 0.79, 3.98), English (*d* = 5.29, 95% CI: 2.76, 7.83), Geography (*d* = 5.29, 95% CI: 2.76, 7.83), Science (*d* = 3.39, 95% CI: 2.62, 4.16) and Arts (*d* = 3.32, 95% CI: 1.84, 4.79). Zinc was found to positively affect English (*d* = 3.78, 95% CI: 0.44, 7.13), Geography (*d* = 4.77, 95% CI: 0.56, 8.98) and Arts (*d* = 2.39, CI: 0.33, 4.45). Iron positively affected Mathematics (*d* = 1.29, 95% CI: 0.54, 2.06), English (*d* = 1.29, 95% CI: 0.44, 7.13), Geography (*d* = 4.77, 95% CI: 0.56, 8.98) and Arts (*d* = 2.39, 95% CI: 0.33, 4.45). *Conclusions*: A more comprehensive intervention with a specific dose/level of physical activity, an increased range of cognitive performance, and a well-designed study design that accounts for dietary intake and other health outcomes are required for future studies.

## 1. Introduction

Cognitive performance is described as the capacity for reasoning, problem-solving, scheduling, nonfigurative judgement, multi-idea comprehension, and experience-based learning and encompasses all elements of general mental competence [1]. It provides critical educational and social experiences for children during their middle childhood stages (ages 6–11 years), and the school years allow children to learn to read, calculate, and develop social skills for interacting with other children and significant adults, as well as acquire broader cultural and social values. Interactions between children and their social and physical environments continue to promote growth [2]. Therefore, it is critical to assist children in maximizing their cognitive potential because these middle childhood stages are one of the crucial periods of brain growth and development [3].

Numerous prior studies have established that factors such as physical activity (PA) and micronutrient intake contribute to children’s cognitive performance. Furthermore, several meta-analyses have demonstrated that physical activity has a beneficial effect on cognitive performance [4,5]. Micronutrients are critical for brain development—vitamins and minerals have been demonstrated to have a significant effect on a variety of physiological processes in the brain and on cognitive performance [6]. Iron, iodine, zinc, folate, vitamin B6, vitamin B12, and vitamin A are necessary for brain growth and, hence, increase children’s cognitive performance [6]. Most published meta-analyses on micronutrients and cognition have centred around only a single type of micronutrient [7,8]. Thus, we performed this review and meta-analysis to assess the effect of physical activity and any micronutrients, alongside the extent of their impact on cognitive performance. This review closely investigated the effects of a range of micronutrients, focusing on how they influence cognition, in addition to the specific duration and method of conducting the intervention. We envisage our findings will aid in the continuation of the studies and strengthen the evidence for the link between the two variables. This review focuses on middle childhood (ages 6–11 years), which is known to be a key period of cognitive development. 

## 2. Materials and Methods

### 2.1. Eligibility Criteria

The formulation of research questions for this study was based on the four main concepts of PICO, specifically Patient/Population, Intervention, Comparison/Control, and Outcome. PICO is a tool that assists authors in developing a suitable research question for review articles. Based on these concepts, the authors have included four main aspects in the meta-analysis, namely children aged 6–11 years (Population), physical activity and micronutrient intake (Intervention), placebo (Comparison), and cognitive performance (Outcome). Studies were considered eligible if they targeted children between the ages of 6 and 11 who did not have any chronic medical condition, mental illness or disability. Participants who received any sort of micronutrient supplementation during pregnancy, infancy or toddlerhood were excluded from this study. The authors evaluated data from randomized controlled trials (RCTs) that examined the impact of physical activity or micronutrients on cognitive performance. The primary and/or secondary results of the RCTs with reporting of at least one of the following cognitive indicators: cognitive performance, academic performance or intelligence quotient (IQ) were selected. Other types of publications, such as review articles, books, book chapters, and conference proceedings, were eliminated due to their status as secondary sources. Language was the third factor for inclusion and exclusion. Only full-text papers published in the English language were included in the study. To minimise misunderstanding and difficulties during the translation process for this review, all non-English language documents were eliminated. Publication years between 2012 and 2021 were chosen to ensure sufficient articles to conduct this review, in accordance with Kraus and colleagues’ definition of ‘research field maturity’ [9]. Additionally, this review used a ten-year time span since it yielded a sufficient number of publications for consideration in the review.

### 2.2. Search Strategy

The present study was drafted according to the Preferred Reporting Items for Systematic reviews and Meta-Analyses (PRISMA) 2020 statement [10]. The protocol for this systematic review and meta-analysis was registered on Open Science Framework (OSF) (registration DOI:10.17605/OSF.IO/EZ8VB). The electronic databases—PubMed (National Library of Medicine and National Institutes of Health of the United States of America) and Scopus^®^ (Elsevier B.V.)—were searched based on the eligibility criteria set. The search strategies combined multiple keyword search terms using Boolean operators. As an illustration, the main keywords used were ‘physical activity’ (keyword 1), ‘micronutrient’ (keyword 2), ‘cognitive performance’ (keyword 3) and ‘children’ (keyword 4). The search method was divided into the following two clusters in addressing two research questions: cluster 1 contained keywords 1 AND 3 AND 4; cluster 2 contained keywords 2 AND 3 AND 4. Physical activity was the primary search phrase (keyword 1), followed by ‘physical activity’ OR ‘exercise’ OR ‘sport’. The key search terms for micronutrient (keyword 2) were ‘vitamin’ OR ‘micronutrient’ OR ‘trace element’ OR ‘minerals’ OR ‘iodine’ OR ‘zinc’ OR ‘iron’ OR ‘vitamin B12’ OR ‘niacin’ OR ‘vitamin B3’ OR ‘vitamin D’ OR ‘folic acid’ OR ‘folate’ OR ‘vitamin D’ OR ‘vitamin D’ OR ‘vitamin C’ OR ‘vitamin B1’ OR ‘vitamin B6’ OR ‘thiamine’ OR ‘vitamin B9’. The key search terms for cognitive performance (keyword 3) were ‘cognitive performance’ OR ‘intelligence quotient’ OR ‘academic performance’ OR ‘academic achievement’. ‘Children’ OR ‘school children’ OR ‘school-aged children’ were the primary search keywords for children (keyword 4). All article screening was conducted independently by two reviewers—A.M.M and A.A. The authors of the publications included in this systematic review were contacted for any missing data. If no response was received, these studies were excluded because they could not be fully assessed for eligibility.

### 2.3. Data Management and Extraction

Mendeley Desktop v1.19.8 (Elsevier, London, UK) was used to import all studies, and duplicates were eliminated using the ‘remove duplicate’ feature. The remaining publications’ titles and abstracts were filtered using the eligibility criteria. The full-text articles were assessed for eligibility, irrelevant publications were eliminated, and quantitative analyses were conducted on studies that fulfilled the inclusion criteria. 

The characteristics of interventions were extracted, including study overview, location of the study, participant, study design, duration of the study, type of the intervention, types of micronutrients and cognitive performance score (attention, Mathematics, English, Geography, Science and Arts). 

### 2.4. Data Quality and Analysis

The Jadad Score for RCTs was used to assess the risk of bias and quality in each study. The score was calculated based on randomization, double-blinding, dropout and withdrawals from trials [11]. The maximum possible score was five, which implies a minimal likelihood of reporting bias, whereas a score of under three was considered at high risk of bias.

The meta-analysis was conducted using random-, fixed- or pooled-effects models with 95% confidence intervals (95% CI). For each kind of cognitive performance, mean intergroup differences were determined by comparing intervention group values to baseline values. Because there were several measurements for evaluating cognitive performance, the most commonly used measurement in the selected studies was chosen. Hence, attention performance and academic subject score (for Mathematics, English, Geography, Science and Arts) were chosen for measuring the children’s cognitive performance. The same concept applied to the type of micronutrients. Only micronutrients with sufficient data were chosen from the selected studies. As a result, this review article focused on vitamin B12, zinc, and iron. Other micronutrients such as iodine, folate, niacin, and thiamine were not included in the present meta-analysis owing to a lack of evidence, i.e., results from the RCT trials. 

The effect size (Cohen’s d) was calculated using an online calculator based on the mean differences and standard deviation (SD) for each cognitive performance (intervention and control groups) [12]. The effect size between groups was considered small (0.2), medium (0.5) and large (0.8). The standard error of the mean (SE) was computed for each outcome measure using the formula SE = es/(es*n), where ‘es’ denotes the effect size. Cochran’s Q and I^2^ were computed automatically using Microsoft Excel spreadsheets [13], which were modified by Ramli et al. after the effect size and SE were inserted [14]. Cochran’s Q was used to validate the existence of heterogeneity in the data, and the I^2^ statistic was used to quantify the amount of heterogeneity. A negative I^2^ value was regarded as comparable to zero (i.e., data were homogeneous), but I^2^ values of 25%, 50% or 75% were considered to have low, medium or high heterogeneity, respectively [15]. The fixed-effects model was chosen for I^2^ values less than 50%, whereas the random-effects model was selected for I^2^ values higher than 50%. The mean effect size data were statistically pooled and shown in a forest plot for the meta-analysis. 

## 3. Results

### 3.1. Study Selection

**Figure 1:**
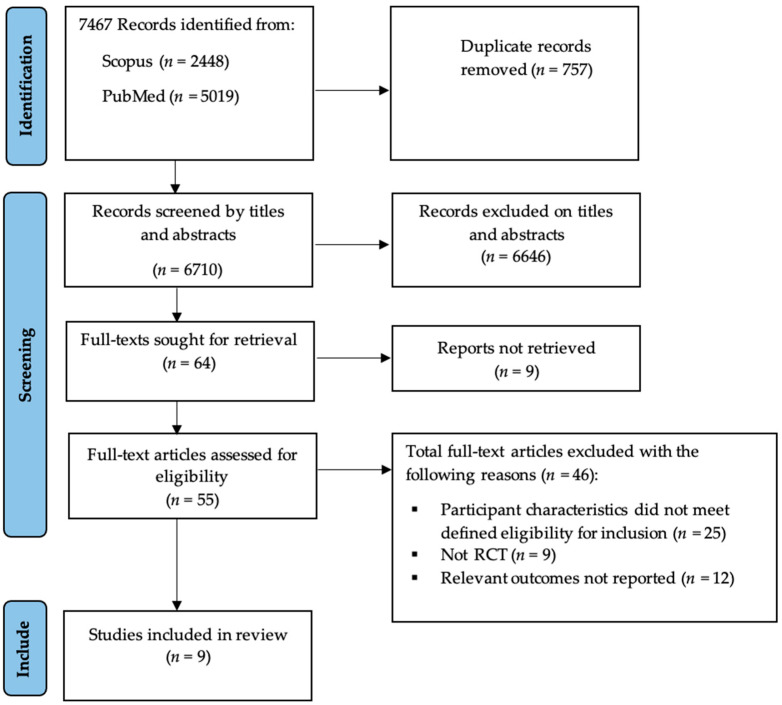
PRISMA flowchart of study selection.

Figure 1 shows the PRISMA flowchart for the study selection process. Our search strategy identified a total of 7467 relevant studies. Seven hundred and fifty-seven duplicate articles were removed, resulting in 6710 unique publications. The titles and abstracts of these 6710 publications were screened according to our eligibility criteria, resulting in the retention of 64 articles. The full text of these 64 articles was retrieved and assessed, resulting in 46 publications being excluded. Nine articles met our selection criteria and were included in our meta-analysis.

### 3.2. Risk of Bias Based on Jadad Score

Table 1 demonstrates the risks of bias of studies based on randomization, double-blinding and dropouts in the RCTs [11]. All studies showed a low risk of bias, with a score of three or more.

### 3.3. Study Characteristics

Table 2 shows the selected 9 RCTs (6 articles) for the relationship between physical activity and cognitive performance. The number of participants in each trial (sample size, n) ranged from 87 to 931 participants, giving a total study population of 2139 participants included in the meta-analysis. There were two types of cognitive performance commonly measured in the RCTs—attention and Mathematics. The duration of the interventions ranged from 4 weeks to 9 months. In the present review, interventions were mostly performed within Europe, including Netherlands, Denmark and Amsterdam. One study was performed in Chile and another one in Australia.

Table 3 shows the findings of five RCTs from three articles regarding the effectiveness of micronutrients on cognitive performance. The number of participants in each trial ranged from 227 to 1190 participants, with a total study participant of 1777. There were six types of cognitive performance commonly measured in the selected RCTs, namely attention, Mathematics, English, Geography, Science and Arts. The types of micronutrients included in the studies were iron, zinc and vitamin B12.

### 3.4. Summary of Meta-Analysis

The outcomes evaluated in this meta-analysis were attention, Mathematics, English, Geography, Science and Arts. Nine RCTs with 2139 participants were included in the meta-analysis for the effect of physical activity on cognitive performance, whereas five RCTs with 1777 participants were included in the meta-analysis for the effect of micronutrients on cognitive performance. Two studies investigated three interventions each and were considered separately in the analyses [16,17]. Mavilidi et al. investigated two types of physical activity intervention, and data from the two groups were treated as findings from two different studies [18].

### 3.5. Effect of Physical Activity on Cognitive Performance

**Figure 2:**
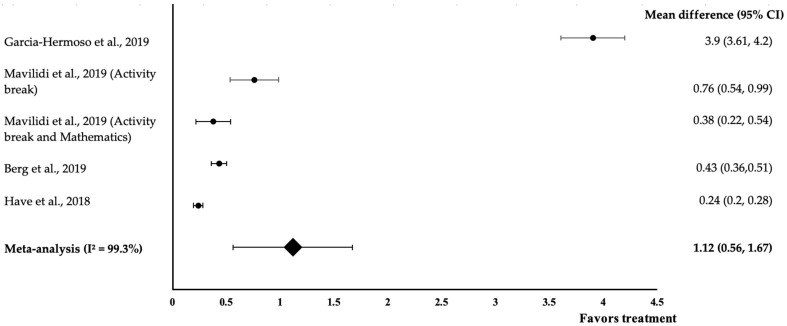
Forest plot showing the effect of physical activity on Mathematics, expressed as mean differences between the values obtained in the intervention and control groups. A positive effect size indicated that physical activity increased Mathematics performance. Horizontal lines represent 95% CIs. Diamonds indicate the pooled-effect size from the random-effects analysis. The values ±0.2, ±0.5, and ±0.8 signify small, medium and large effect sizes, respectively [18,19,20,21].

Two different types of cognitive performance were measured in this analysis—Mathematics and attention. Figure 2 shows the meta-analysis of the effect of physical activity on Mathematics of four studies with five trials. All five interventions showed positive effects on Mathematics (treatment group favoured). The study by Garcia-Hermoso et al. demonstrated the largest effect size (*d* = 3.9, 95% CI: 3.61, 4.2), followed by Mavilidi et al. (Activity break) (*d* = 0.76, 95% CI: 0.54, 0.99), Berg et al. (*d* = 0.43, 95% CI: 0.36,0.51), Mavilidi et al. (Activity break and Mathematics) (*d* = 0.38, 95% CI: 0.22, 0.54) and Have et al. (*d* = 0.24, 95% CI: 0.2, 0.28). The data showed a high level of heterogeneity with I^2^ = 99.3% and, hence, were subjected to random-effects analysis. Physical activity exhibited a large pooled-effect size on Mathematics, with *d* = 1.12 (95% CI: 0.56, 1.67). 

**Figure 3:**
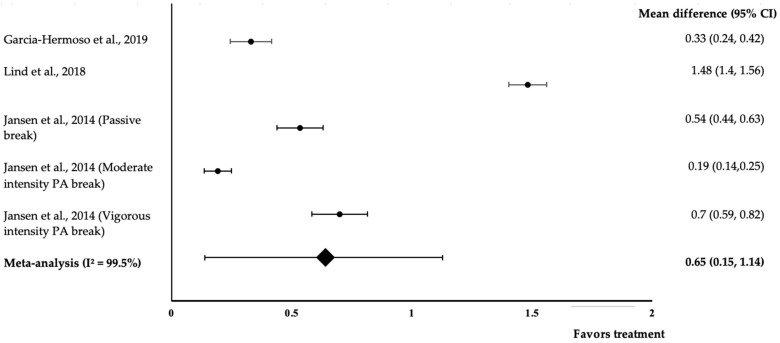
Forest plot showing the effect of physical activity on attention, expressed as mean differences between the values obtained in the intervention and control groups. A positive effect size indicated that physical activity increased attention. Horizontal lines represent 95% CIs. Diamonds indicate the pooled-effect size from the random-effects analysis. The values ±0.2, ±0.5, and ±0.8 are illustrative of small, medium and large effect sizes, respectively [16,19,22].

Figure 3 shows the meta-analysis of the three studies with five trials that aimed to improve cognitive performance via physical activity interventions that were included. All five interventions exhibited positive effects on attention (treatment group favoured). Lind and co-workers’ study showed the highest effect size (*d* = 1.48, 95% CI: 1.4, 1.56), followed by Janssen et al. (vigorous intensity PA break) (*d* = 0.7, 95% CI: 0.54, 0.82), Janssen et al. (passive break) (*d* = 0.54, 95% CI: 0.44,0.63), Garcia-Hermoso et al. (*d* = 0.33, 95% CI: 0.24) and Janssen et al. (moderate intensity PA break) (*d* = 0.19, 95% CI: 0.14, 0.25). The data showed a high level of heterogeneity with I^2^ = 99.5% and, hence, were subjected to random-effects analysis. Physical activity demonstrated a medium pooled-effect size on attention, with a *d* value of 0.65 (95% CI: 0.15, 1.14). 

### 3.6. Effect of Micronutrients on Cognitive Performance

**Figure 4:**
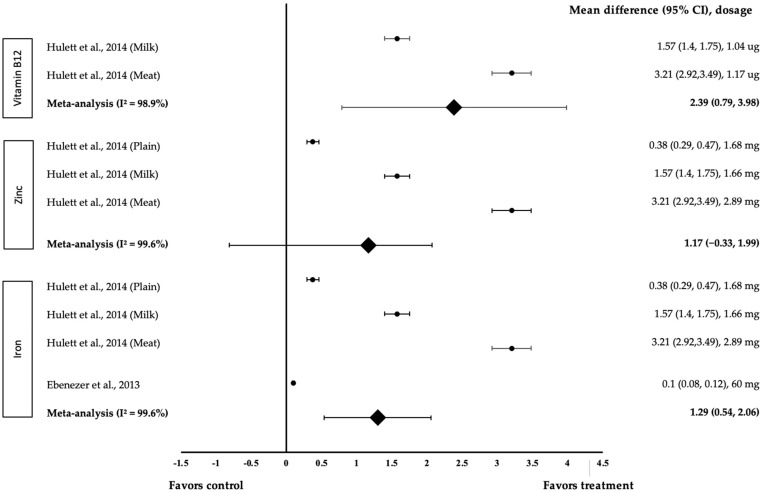
Forest plot showing the effect of vitamin B12, zinc and iron on Mathematics, expressed as mean differences between the values obtained in the intervention and control groups. A positive effect size indicated that vitamin B12, zinc and iron increased Mathematics skills. Horizontal lines represent 95% CIs. Diamonds indicate the pooled-effect size from the random-effects analysis. The values ±0.2, ±0.5 and ±0.8 represent small, medium and large effect sizes, respectively [17,23].

Six different types of cognitive performance were measured in this analysis—Mathematics, English, Geography, Science, Arts and attention. Three types of micronutrients were measured in the meta-analysis, namely vitamin B12, iron and zinc. Figure 4 shows the meta-analysis of the impact of micronutrients on Mathematics. Two studies with four trials were included in the meta-analysis. For vitamin B12, both interventions showed a positive effect on Mathematics (treatment group favoured). The study by Hullet et al. showed that 1.17 µg of B12 in meat Githeri have the highest effect size (*d* = 3.21, 95% CI: 2.92, 3.49), followed by 1.04 µg of B12 in milk Githeri (*d* = 1.57, 95% CI: 1.4, 1.75). The data showed a high level of heterogeneity with I^2^ = 98.9% and, hence, were subjected to random-effects analysis. Vitamin B12 demonstrated a large pooled-effect size on Mathematics, with a *d* value of 2.39 (95% CI: 0.79, 3.98). With zinc, all three interventions showed positive effects on Mathematics (treatment group favoured). 2.89 mg of zinc in meat Githeri provided the highest effect size (*d* = 3.21, 95% CI: 2.92, 3.49), followed by 1.66 mg of zinc in milk Githeri (*d* = 1.57, 95% CI: 1.4, 1.75) and 1.68 mg of zinc in plain Githeri (*d* = 0.38, 95% CI: 0.29, 0.47). The data showed a high level of heterogeneity with I^2^ = 99.6% and, therefore, were subjected to random-effects analysis. Zinc showed a large pooled-effect size on Mathematics, with *d* = 1.17 (95% CI: −0.9, 1.99). As for iron, all four interventions exhibited a positive effect on Mathematics (treatment group favoured). 2.94 mg of iron in meat Githeri provided the highest effect size (*d* = 3.21, 95% CI: 2.92, 3.49), followed by 1.57 mg of iron in milk Githeri (*d* = 1.57, 95% CI: 1.4, 1.75), 3.93 mg of iron in plain Githeri (*d* = 0.38, 95% CI: 0.29, 0.47) and Ebenezer et al. with 60 mg of iron (*d* = 0.1, 95% CI: 0.08, 0.12). The data showed a high level of heterogeneity with I^2^ = 99.6%; hence, these data were subjected to random-effects analysis. Iron demonstrated a large pooled-effect size on mathematics, with *d* = 1.29 (95% CI: 0.54, 2.06).

**Figure 5:**
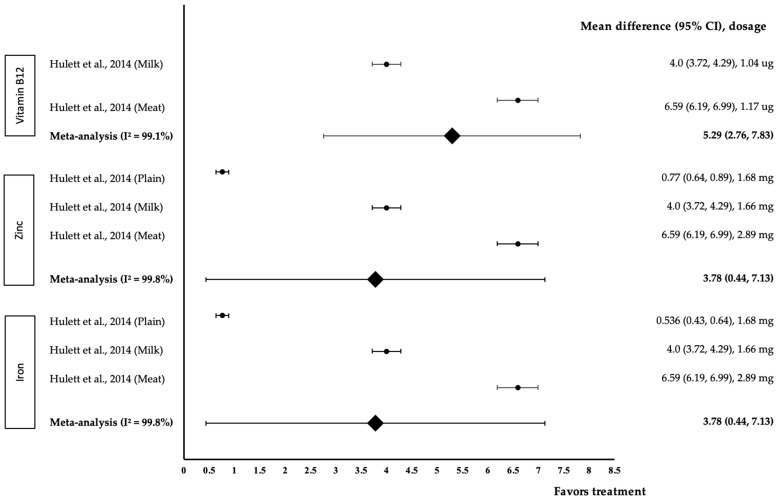
Forest plot illustrating the effect of vitamin B12, zinc and iron on English, expressed as mean differences between the values obtained in the intervention and control groups. A positive effect size indicated that vitamin B12, zinc and iron increased English skills. Horizontal lines represent 95% CIs. Diamonds are indicative of the pooled-effect size from the random-effects analysis. The values ±0.2, ±0.5, and ±0.8 represent small, medium and large effect sizes, respectively [17].

Figure 5 shows the meta-analysis of the effect of micronutrients on English. One study with three trials was included in the meta-analysis. As for the effect of vitamin B12, both interventions were associated with a positive effect on English (treatment group favoured). The work of Hullet et al. with 1.17 µg of B12 in meat Githeri demonstrated the highest effect size (*d* = 6.56, 95% CI: 6.19, 6.99), followed by 1.04 µg of B12 in milk Githeri (*d* = 4.0, 95% CI: 3.72, 4.29), respectively. The data showed a high level of heterogeneity with I^2^ = 99.1% and were, therefore, subjected to random-effects analysis. Vitamin B12 showed a large pooled-effect size on English, with *d* = 5.29 (95% CI: 2.76, 7.83). As for the effect of zinc, all three interventions displayed a positive effect on English (treatment group favoured). Zinc (2.89 mg) in meat Githeri showed the highest effect size (*d* = 6.59, 95% CI: 6.19, 6.55), followed by 1.66 mg of zinc in milk Githeri (*d* = 4.0, 95% CI: 3.72, 4.29) and 1.68 mg of zinc in plain Githeri (*d* = 0.77, 95% CI: 0.64, 0.89). The data demonstrated a high level of heterogeneity with I^2^ = 99.8%; thus, these data were subjected to random-effects analysis. Zinc was associated with a large pooled-effect size on English, with *d* = 3.78 (95% CI: 0.44, 7.13). Regarding the effect of iron, all three interventions produced a positive effect on English (treatment group favoured). Iron (2.94 mg) in meat Githeri showed the highest effect size (*d* = 6.59, 95% CI: 6.19, 6.55), followed by 1.57 mg of iron in milk Githeri (*d* = 4.0, 95% CI: 3.72, 4.29) and 3.93 mg of iron in plain Githeri (*d* = 0.77, 95% CI: 0.64, 0.89). The data demonstrated a high level of heterogeneity with I^2^ = 99.6% and, therefore, were subjected to random-effects analysis. Iron showed a large pooled-effect size on English, with *d* = 1.29 (95% CI: 0.44, 7.13).

**Figure 6:**
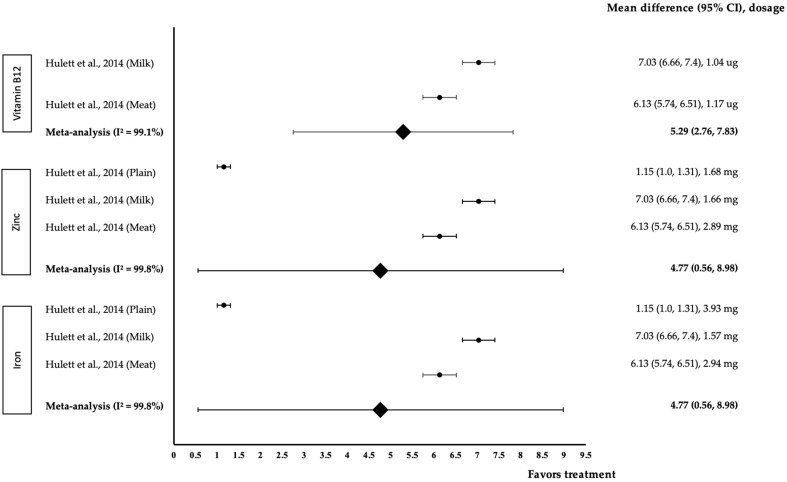
Forest plot showing the effect of vitamin B12, zinc and iron on Geography, expressed as mean differences between the values obtained in the intervention and control groups. A positive effect size indicated that vitamin B12, zinc and iron increased Geography skills. Horizontal lines represent 95% CIs. Diamonds indicate the pooled-effect size from the random-effects analysis. The values ±0.2, ±0.5 and ±0.8 represent small, medium and large effect sizes, respectively [17].

Figure 6 shows the meta-analysis of the effect of micronutrients on Geography. One study with three trials was included in the meta-analysis. As for the effect of vitamin B12, both interventions showed a positive effect on Geography (treatment group favoured). Hullet and colleagues’ study with 1.04 µg of B12 in milk Githeri provided the highest effect size (*d* = 7.03, 95% CI: 6.66, 7.4), followed by 1.17 µg of B12 in meat Githeri (*d* = 6.13, 95% CI: 5.74, 6.51). The data showed a high level of heterogeneity with I^2^ = 99.1% and, hence, were subjected to random-effects analysis. Vitamin B12 demonstrated a large pooled-effect size on Geography, with *d* = 5.29 (95% CI: 2.76, 7.83). Regarding zinc’s effect, all three interventions were found to positively affect Geography (treatment group favoured). 1.66 mg of zinc in milk Githeri provided the highest effect size (*d* = 7.03, 95% CI: 6.66, 7.4), followed by 2.89 mg of zinc in meat Githeri (*d* = 6.13, 95% CI: 5.74, 6.51) and 1.68 mg of zinc in plain Githeri (*d* = 1.15, 95% CI: 1.0, 1.31). The data showed a high level of heterogeneity with I^2^ = 99.8%, and hence, were subjected to random-effects analysis. Zinc was associated with a large pooled-effect size on Geography, with *d* = 4.77 (95% CI: 0.56, 8.98). In terms of the effect of iron, all three interventions positively affected Geography (treatment group favoured). Iron (1.57 mg) in milk Githeri produced the highest effect size (*d* = 7.03, 95% CI: 6.66, 7.4), followed by 2.94 mg of iron in meat Githeri (*d* = 6.13, 95% CI: 5.74, 6.51) and 3.93 mg of iron in plain Githeri (*d* = 1.15, 95% CI: 1.0, 1.31). The data showed a high level of heterogeneity with I^2^ = 99.8%; therefore, these data were subjected to random-effects analysis. Iron showed a large pooled-effect size on geography, with *d* = 4.77 (95% CI: 0.56, 8.98).

**Figure 7:**
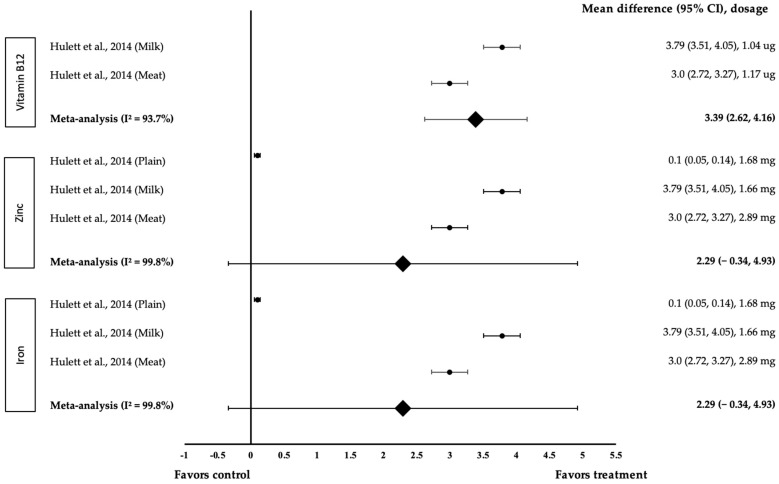
Forest plot showing the effect of vitamin B12, zinc and iron on Science, expressed as mean differences between the values obtained in the intervention and control groups. A positive effect size indicated that vitamin B12, zinc and iron increased Science skills. Horizontal lines represent 95% CIs. Diamonds indicate the pooled-effect size from the random-effects analysis. The values ±0.2, ±0.5, and ±0.8 represent small, medium and large effect sizes, respectively [17].

Figure 7 shows the meta-analysis of the effect of micronutrients on Science. One study with three trials was included in the meta-analysis. As for the effect of vitamin B12, both interventions showed a positive effect on Science (treatment group favoured). Hullet and co-workers’ research with 1.04 µg of B12 in milk Githeri demonstrated the highest effect size (*d* = 3.79, 95% CI: 3.51, 4.05), followed by 1.17 µg of B12 in meat Githeri (*d* = 3.0, 95% CI: 3.51, 4.05). The data were associated with a high level of heterogeneity with I^2^ = 93.7% and were, therefore, subjected to random-effects analysis. Vitamin B12 showed a large pooled-effect size on Science, with *d* = 3.39 (95% CI: 2.62, 4.16). As for the effect of zinc, all three interventions showed a positive effect on Science (treatment group favoured). Zinc (1.66 mg) in milk Githeri demonstrated the highest effect size *d* = 3.79, 95% CI: 3.51, 4.05, followed by 2.89 mg of zinc in meat Githeri (*d* = 3.0, 95% CI: 3.51, 4.05) and 1.68 mg of zinc in plain Githeri (*d* = 0.1, 95% CI: 0.05, 0.14). The data showed a high level of heterogeneity with I^2^ = 99.8% and, hence, were subjected to random-effects analysis. Zinc exhibited a large pooled-effect size on Science, with *d* = 2.29 (95% CI: −0.34, 4.93). Regarding iron’s effect, all three interventions were associated with a positive effect on Science (treatment group favoured). Iron (1.57 mg) in milk Githeri provided the highest effect size (*d* = 3.79, 95% CI: 3.51, 4.05), followed by 2.94 mg of iron in meat Githeri (*d* = 3.0, 95% CI: 3.51, 4.05) and 3.93 mg of iron in plain Githeri (*d* = 0.1, 95% CI: 0.05, 0.14). The data showed a high level of heterogeneity with I^2^ = 99.8%; these data were, therefore, subjected to random-effects analysis. Iron demonstrated a large pooled-effect size on science, with *d* = 2.29 (95% CI: −0.34, 4.93).

**Figure 8:**
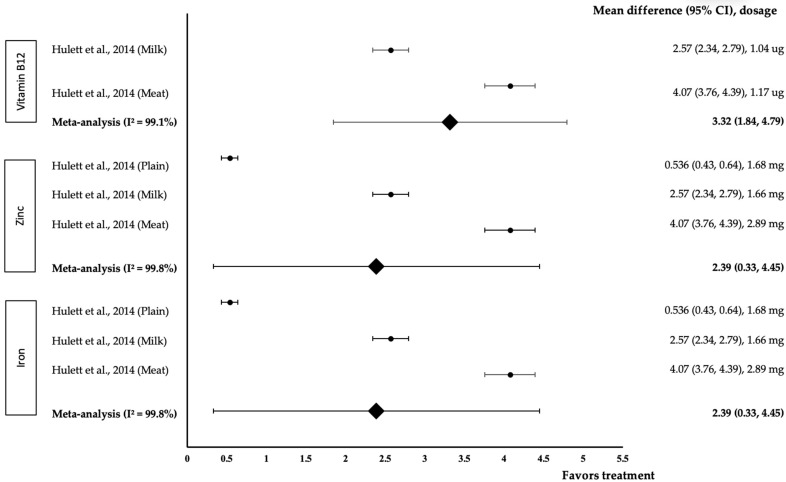
Forest plot showing the effect of vitamin B12, zinc and iron on Arts, expressed as mean differences between the values obtained in the intervention and control groups. A positive effect size indicated that vitamin B12, zinc and iron increased Arts capabilities. Horizontal lines represent 95% CIs. Diamonds indicate the pooled-effect size from the random-effects analysis. The values ±0.2, ±0.5, and ±0.8 represent small, medium and large effect sizes, respectively [17].

Figure 8 shows the meta-analysis of the effect of micronutrients on Arts. One study with three trials was included in the meta-analysis. As for the effect of vitamin B12, both interventions showed a positive effect on Arts (treatment group favoured). The study by Hullet et al. with 1.17 µg of B12 in meat Githeri demonstrated the highest effect size (*d* = 4.07, 95% CI: 3.76, 4.39), followed by 1.04 µg of B12 in milk Githeri (*d* = 2.57, 95% CI: 2.34, 2.79). The data showed a high level of heterogeneity with I^2^ = 99.1% and, hence, were subjected to random-effects analysis. Vitamin B12 showed a large pooled-effect size on Arts, with *d* = 3.32 (95% CI: 1.84, 4.79). In terms of the effect of zinc, all three interventions positively affected Arts (treatment group favoured). Zinc (2.89 mg) in meat Githeri showed the highest effect size (*d* = 4.07, 95% CI: 3.76, 4.39), followed by 1.66 mg of zinc in milk Githeri (*d* = 2.57, 95% CI: 2.34, 2.79) and 1.68 mg of zinc in plain Githeri (*d* = 0.54, 95% CI: 0.43, 0.64). The data showed a high level of heterogeneity with I^2^ = 99.8%; thus, they were subjected to random-effects analysis. Zinc showed a large pooled-effect size on Arts, with *d* = 2.39 (95% CI: 0.33, 4.45). As for the effect of iron, all three interventions produced a positive effect on Arts (treatment group favoured). Iron (2.94 mg) in meat Githeri provided the highest effect size (*d* = 4.07, 95% CI: 3.76, 4.39), followed by 1.57 mg of iron in milk Githeri (*d* = 2.57, 95% CI: 2.34, 2.79) and 3.93 mg of iron in plain Githeri (*d* = 0.54, 95% CI: 0.43, 0.64). Therefore, the data showed a high level of heterogeneity with I^2^ = 99.8% and were subjected to random-effects analysis. Iron provided a large pooled-effect size on arts, with *d* = 2.39 (95% CI: 0.33, 4.45).

**Figure 9:**
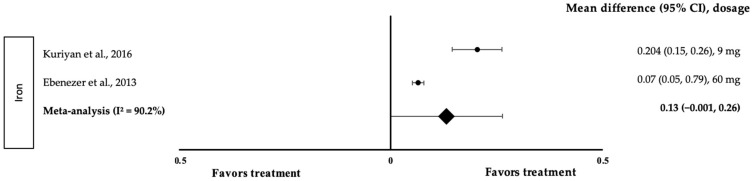
Forest plot showing the effect of iron on attention, expressed as mean differences between the values obtained in the intervention and control groups. A positive effect size indicated that iron increased attention. Horizontal lines represent 95% CIs. Diamonds signify the pooled-effect size from the random-effects analysis. The values ±0.2, ±0.5 and ±0.8 indicate small, medium and large effect sizes, respectively [23,24].

Figure 9 indicates the meta-analysis of the effect of iron on attention, which included two studies with two trials. Both interventions were shown to positively affect attention (treatment group favoured). Kuriyan and colleagues’ study with 9 mg of iron was associated with the highest effect size (*d* = 0.204, 95% CI: 0.15, 0.26), followed by Ebenezer et al. with 60 mg of iron (*d* = 0.07, 95% CI: 0.05, 0.79). Because the data demonstrated a high level of heterogeneity with I^2^ = 90.2%, they were subjected to random-effects analysis. Iron displayed a small pooled-effect size on attention, with a *d* value of 0.13 (95% CI: −0.001, 0.26).

## 4. Discussion

This systematic review with meta-analysis synthesized the evidence of the effectiveness of physical activity and micronutrients in increasing children’s cognitive performance. It was evident that physical activity had a substantial impact on both Mathematics and attention. However, Mathematics showed a larger pooled effect size compared to attention. Hence, this review suggested that physical activity had a higher effect on increasing Mathematics scores compared to the level of attention. This observation might be due to the duration of the intervention that the longer the intervention, the more likely attention capacity will improve [19]. According to work by Lind and colleagues’, the effect of physical activity on attention had the largest effect size, and their study had the longest intervention period among all five treatments at 11 weeks [22]. This result can be supported by a previous meta-analysis by de Greeff et al., where longitudinal physical activity programs positively affect cognitive functions as well as academic performance compared to physical activity break interventions [25]. In line with our findings, their results suggested an association between the duration of the physical activity program and improved cognitive performance. Besides the length of intervention, the type of physical activity can also influence cognitive performance. Physical activity programs, such as the Active-Start program [19], can show more improvement compared to physical activity breaks, as conducted by Mavillidi et al. and Have et al. with respect to Mathematics performance [18,20]. In support of these findings, a previous meta-analysis also found to demonstrate that physically active lessons are more effective than active breaks [5].

Program design and teaching strategies can also influence the effectiveness of an intervention. Alvarez-Bueno et al. found that interventions developed by a trained specialist are associated with increased benefits [4]. Based on our analysis of the studies, Garcia-Hermoso et al. and Lind et al. had the most effect on cognitive performance (Figure 2 and Figure 3). The intervention programs in both studies were developed and carried out by trained specialists, such as in the ‘FIFA 11 for Health’ for Europe program run by staff from the University of Southern Denmark and football coaches from the Danish Football Association [22]. The Active-Start intervention was designed by the research team and delivered by a graduate in Sport Sciences [19]. Contrasting with the other three studies [18,20,21], the intervention was carried out by a classroom teacher who had a lower qualification. This teacher might not have been aware or sufficiently alert whether they made a mistake and simply followed the instructions given by the researcher during the deliveries of the intervention. This finding can be further supported by Sember et al., who found that interventions performed by staff with higher qualifications are more effective compared to those administered by practitioners with lower qualifications in the field [26]. 

Based on our meta-analysis, it is evident that micronutrients had a substantial impact on several cognitive performance areas, such as Mathematics, English, Geography and Arts. Micronutrients, such as vitamin B12, zinc and iron met the inclusion criteria and hence, they were included in the analysis [17,23,24]. These three nutrients are essential for brain development and are postulated to influence cognitive performance. Iron is involved in oligodendrocyte development and myelin production [27] and is a cofactor for neurotransmitter synthesis [28]. Zinc is associated with neuronal migration, synaptogenesis and neurogenesis [29]. Vitamin B12 affects methylation in the central nervous system [30] and maintains the integrity of the myelin sheath via vascular disease prevention [31]. Based on our meta-analysis, iron and vitamin B12 had a substantial effect on Mathematics performance. Furthermore, vitamin B12 had the largest pooled effect size and is, therefore, more effective than zinc and iron. A larger dose of vitamin B12 has also been shown to improve Mathematics performance. Iron, on the other hand, was not as effective. According to Ebenezer et al., even though the dosage was the largest compared to the other treatments, there was no effect on cognitive performance [23]. Aside from that, the three Hullet et al. treatments used a snack that may have included different nutrients from the supplement used in the Ebenezer et al. trial [17,23]. The effect of vitamin B12, zinc and iron on English and Arts has been studied in a comparative meta-analysis (Figure 5 and Figure 8). However, as compared to Arts performance, English performance had a larger pooled-effect size for each of the three micronutrients. The dose of vitamin B12 and zinc may have influenced English and Arts performance because the higher the dosage, the larger the effect size. A 1.17 µg of vitamin B12 has been found to be more effective than 1.04 µg, and 2.94 mg of zinc is more effective than 1.68 mg and 1.66 mg. However, iron supplementation does not appear to influence English or Arts performance [17]. Vitamin B12, zinc and iron have a major impact on Geography performance. Geography test results improved considerably with increased consumption of iron, calories per kg of body weight, vitamin B12, zinc and riboflavin [17]. In terms of the link between micronutrients and Science performance, only vitamin B12 had a significant effect. The consumption of other nutrients (energy, protein, iron, zinc, folate, vitamin B6, riboflavin) was not associated with Science test scores [17]. 

This study did not find a significant association between iron and attention in the meta-analysis as the error bar was close to the centre line (*d* = 0.13, 95% CI: −0.001, 0.26). This observation is supported by Gou et al., who claimed that iron did not improve global cognitive scores [8]. However, a contradictory result was provided by Low et al., who observed that iron could improve the global cognitive score and the measures of attention [32]. Kuriyan et al. showed a higher effect compared to Ebenezer et al., potentially because multi-nutrient-fortified foods or drinks are more beneficial than supplements [23,24]. A multiple micronutrient food supplement (MMFS) added to school meals daily for 12 months was found to significantly improve attention [33]. Kuriyan et al. used fortified milk as the intervention in their study [24], an approach that is more likely to be helpful because it may also include other nutrients that aid in cognitive growth. According to one study, 0.9 mg of zinc and 0.54 µg of vitamin B12 improved children’s attention when added together in milk [24]. A combination of a few nutrients may be more effective in increasing cognitive development compared to a single nutrient. Hence, it is suggested that multi-nutrient intervention is likely associated with a positive effect on cognitive performance.

This review found the effects of three micronutrients (vitamin B12, iron and zinc) on cognitive performance. Other important micronutrients, namely iodine, folate, vitamin B6, and vitamin A were not included in this review due to poor-quality data. Further RCTs investigating the effect of these micronutrients on cognitive performance are highly encouraged, especially in children aged 6 to 11 Years.

## 5. Conclusions

Collectively, our review and meta-analysis comprised nine articles with 14 high-quality RCTs, nine of which focused on physical activity and five on micronutrients with respect to cognitive performance. It is evident that both physical activity and micronutrients had substantial impacts on cognitive performance. The intervention duration was found to be important because longer research periods were sufficient for evaluating significant improvements in cognitive performance, particularly attention. According to the findings of our meta-analysis, physical activity influenced both Mathematics and attention performance. In terms of micronutrients, vitamin B12 had an impact on Mathematics, English, Geography, Science and Arts. Zinc influenced English, Geography and Arts, whereas iron affected Mathematics, English, Geography and Arts. The study data showed a significant level of heterogeneity; hence, conclusions shall be taken with care. To further validate the findings described in this review, a more comprehensive intervention with a particular dose, degree of physical activity, a higher range of cognitive performance and a well-planned research study design, with adjustment for dietary intake and other health outcomes, are required in the future.

## Figures and Tables

**Table 1:** Summary of assessment of the risk of bias (*n* = 9).

Studies	Randomization (yes/no)	Appropriateness of Randomization (Detail)	Blinding (yes/no) *	Appropriateness of Blinding	An account of all Participants or Description of Withdrawal or Dropouts	Total Score
Garcia-Hermoso et al. (2019)	1	1	N/A	N/A	1	3
Mavilidi et al. (2019)	1	1	N/A	N/A	1	3
Berg et al. (2019)	1	1	N/A	N/A	1	3
Have et al. (2018)	1	1	0.5	1	1	4.5
Lind et al. (2018)	1	1	N/A	N/A	1	3
Kuriyan et al. (2016)	1	1	1	1	1	5
Janssen et al. (2014)	1	1	N/A	N/A	1	3
Hulett et al. (2014)	1	1	N/A	N/A	1	3
Ebenezer et al. (2013)	1	1	1	1	1	5

* Double blinded = 1 point; single blinded = 0.5 point; N/A: Not available.

**Table 2:** Summary of randomized controlled trials for the effectiveness of physical activity (PA) on cognitive performance.

Study	Participant	Location	Study Design	Staff Implementing Intervention and Measurements (HQ or LQ)	Type of PA Intervention	Cognitive Performance *	
						Attention	Mathematics
Garcia-Hermoso et al. (2019)	70 children (8–10 years old)	Chile	Randomized, non-blinded, parallel design IG: 100 CG: 70 Duration: 8 weeks	Graduates in Sport Sciences (HQ)	The Active-Start program (i.e., program of cooperative physical games) was structured to make group cooperation essential to game success and to encourage pro-social skills.	IG = 62.48 ± 6.58 CG = 60.15 ± 7.66 *p*-value = 0.124 (effect size = 0.331)	IG = 0.02 ± 0.1 CG = 0.48 ± 0.16 *p* < 0.001 (effect size = 3.903)
Mavilidi et al. (2019)	87 children (9–10 years old)	Australia	Randomized, non-blinded, parallel design IG_1_: 29 IG_2_: 29 CG: 29 Duration: 4 weeks	Classroom teacher (LQ)	IG_1_: The activity breaks condition—divided into two minutes of activity break at the beginning of the lesson and three minutes in the middle of the lesson.	N/A	IG_1_ = 0.19 ± 2.55 CG = 2.14 ± 2.57 *p* = 0.045 (effect size = 0.762)
					IG_2_: Activity breaks and Mathematics combined condition—Students performed the PA shown in the video while they answered the mathematical questions.	N/A	IG_2_ = 3.11 ± 2.55 CG = 2.14 ± 2.57 *p* = 0.185 (effect size = 0.379)
Berg et al. (2019)	323 children (10–11 years old)	Netherlands	Cluster-randomized controlled trial, non-blinded, parallel design IG: 170 CG: 153 Duration: 5 weeks	Classroom teacher (LQ)	Juggling exercises—week 1 and 2, two balls in week 3 and 4, and ending with using three balls in week 5 of the program.	N/A	IG = 25.9 ± 4.8 CG = 23.3 ± 7.1 (effect size = 0.433)
Have et al. (2018)	505 children (7–8 years old)	Denmark	Cluster-Randomized, single-blinded, parallel design IG: 294 CG: 211 Duration: 9 months	Classroom teacher (LQ)	15–20 min of PA spread over an average of 6 mathematics lessons of 45 min per week	N/A	IG_1_ = 1.2 ± 6.56 CG = 0 (effect size = 0.240)
Lind et al. (2018)	931 children (11 years old)	Denmark	Cluster-randomized, non-blinded, parallel design IG: 93 CG: 838 Duration: 11 weeks	Staff from the University of Southern Denmark and football coaches from the Danish Football Association (HQ)	FIFA 11 for Health for Europe—consisted of two 45-min football sessions, totalling 990 min over the 11 weeks	IG = 598.54 ± 5.54 CG = 618.19 ± 13.85 (effect size = 1.482)	N/A
Janssen et al. (2014)	123 children (10–11 years old)	Amsterdam	Randomized, non-blinded, parallel design IG_1_: 108 IG_2_: 111 IG_3_: 89 CG: 112	Researchers (HQ)	IG_1_: Passive break	IG_1_ = 2.5 ± 0.71 CG = 2.9 ± 0.78 (effect size = 0.536)	N/A
					IG_2_: Moderate intensity PA break	IG_2_ = 2.1 ± 5.8 CG = 2.9 ± 0.78 (effect size = 0.194)	N/A
					IG_3_: Vigorous intensity PA break	IG_3_ = 2.4 ± 0.62 CG = 2.9 ± 0.78 (effect size = 0.701)	N/A

* Values are the mean ± SD. Abbreviations: IG, intervention group; CG, control group; PA, physical activity; FIFA, Federation Internationale de Football Association; HQ, staff with higher professional qualifications; LQ, staff with lower professional qualifications; N/A, not available.

**Table 3:** Summary of effectiveness of randomized controlled trials (RCTs) for micronutrients on cognitive performance.

Study	Study Overview	Study Design	Type of Micronutrient and Doses	Cognitive Performance *					
				Mathematics	English	Geography	Science	Arts	Attention
Hulett et al. (2014)	Subject: 360 children (7–8 years old) Country: Kenya	Cluster-randomized, non-blinded, controlled feeding intervention trial, parallel design Treatment: (1) Plain Githeri (*n* = 99) (2) Githeri + Milk (*n* = 105) (3) Githeri + Meat (*n* = 67) (4) Control (*n* = 89) Duration: two years	(1) Plain Githeri Iron = 3.93 mg Zinc = 1.68 mg	IG = 2.48 ± 1.81 CG = 3.2 ± 2.0 (effect size = 0.378)	IG = −7.51 ± 2.36 CG = −9.32 ± 2.36 (effect size = 0.765)	IG = −1.52 ± 2.36 CG = −3.88 ± 1.63 (effect size = 1.153)	IG = −6.96 ± 1.82 CG = −6.78 ± 1.82 (effect size = 0.099)	IG = 4.29 ± 1.27 CG = 3.56 ± 1.46 (effect size = 0.536)	N/A
			(2) Githeri + Milk Iron = 1.57 mg Zinc = 1.66 mg vitamin B12 = 1.04 µg	IG = 5.92 ± 1.46 CG = 3.2 ± 2.0 (effect size = 1.574)	IG = −0.97 ± 1.82 CG = −9.32 ± 2.36 (effect size = 4.005)	IG = 6.29 ± 1.27 CG = −3.88 ± 1.63 (effect size = 7.032)	IG = -0.61 ± 1.45 CG = −6.78 ± 1.82 (effect size = 3.785)	IG = 6.83 ± 1.09 CG = 3.56 ± 1.46 (effect size = 2.569)	N/A
			(3) Githeri + Meat Iron = 2.94 mg Zin = 2.89 mg vitamin B12 = 1.17 µg	IG = 9.37 ± 1.82 CG = 3.2 ± 2.0 (effect size = 3.205)	IG = 5.74 ± 2.18 CG = −9.32 ± 2.36 (effect size = 6.592)	IG = 6.11 ± 1.63 CG = −3.88 ± 1.63 (effect size = 6.129)	IG = −1.34 ± 1.81 CG = −6.78 ± 1.82 (effect size = 2.996)	IG = 9.19 ± 1.27 CG = 3.56 ± 1.46 (effect size = 4.074)	N/A
Kuriyan et al. (2016)	Subject: 227 children (7–10 years old) Country: India	Randomized, double blind, placebo-controlled study, parallel design Treatment: (1) Fortified Milk (*n* = 111) (2) Control (*n* = 114) Duration: 5 months	(1) Iron = 18 mg/2 serving (2) Zinc = 1.8 mg/2 serving (3) Vitamin B12 = 1.08 mcg/2 serving	N/A	N/A	N/A	N/A	N/A	IG = 2.0 ± 0.57 CG = 1.9 ± 0.4 (effect size = 0.204)
Ebenezer et al. (2013)	Subject: 1190 (8–10 years old) Country: Sri Lanka	Prospective, placebo-controlled randomized, parallel design Treatment: (1) Iron Supplement (*n* = 615) (2) Control (*n* = 575) Duration: 6 months	Iron = 60 mg	IG = 13.9 ± 17.4 CG = 12.2 ± 16.1 (effect size = 0.101)	N/A	N/A	N/A	N/A	IG = 3.4 ± 6.1 CG = 3.0 ± 6.3 (effect size = 0.065)

* Values are the mean ± SD; Abbreviations: IG, intervention group; CG, control group; N/A, not available.

## Data Availability

The data presented in this study are available upon request of the corresponding author.
